# Eosionphilic oesophagitis in Cape Town, South Africa

**DOI:** 10.1186/2045-7022-1-S1-P26

**Published:** 2011-08-12

**Authors:** Michael Levin, Cassim Motala

**Affiliations:** 1 University of Cape Town, Division of Allergy, Paediatrics and Child Health, Cape Town, South Africa

## 

Eosinophilic oesophagitis has been described in patients from all ethnic backgrounds in studies originating in all continents apart from Africa.

A cohort of 8 patients (3 boys, 5 girls) identified at Red Cross Hospital during 2009-2010 is described. Average age 7 years (1yr 11 months to 15 years 10 months). Ethnicity 2 caucasian, 5 mixed, 1 Black African. Age of onset: mean 3 years, median 1 year 4 months. Age of diagnosis mean 6years 3 months, median 3 years 9 months.

Time to diagnosis: mean 3 years 3 months, median 6 months, IQ range 5 months to 6 years.

Presenting symptoms in order of prevalence are reflux (7/8), long time to eat (6/8), difficult swallowing (6/8), growth failure (5/8), food refusal (5/8) and painful swallowing (4/8).

Associated atopic diseases comprised immediate food allergy (6/8), eczema (6/8), rhinitis (6/8), asthma (3/8) and urticaria (2/8).

Total of 26 biopsy specimens, mean 3.25 per patient. Only 4/8 confirmed peak eosinophil count >15/hpf, 7/8 had minor features present.

Food skin prick tests 152 (19 per patient). Positive skin tests >=1mm 57 (13 per patient). The most commonly identified foods are peas, wheat, milk, egg white, banana and egg yolk.

Skin tests >=3mm 32 (7 per patient). Most commonly identified foods by SPT >3mm are egg yolk, egg white, peas, soya, rye, rice, carrot and green beans.

Patch tests 167 (21 per patient). 30 positive, average of 4.3 per patient. Most commonly identified foods are beef, peanut, lamb, chicken, soy and ham.

All commenced on initiation of short course of oral steroids. All put on targeted elimination diet. All had clinical improvement. 3 controlled and acceptable symptoms, 2 some symptoms and difficulties, 2 very symptomatic with poor control, 1 defaulted.

